# Gerstmann Syndrome in an Elderly Patient: A Case Report Presented with a Complete Tetrad of Symptoms

**DOI:** 10.3390/medicina60101640

**Published:** 2024-10-07

**Authors:** Corneliu Toader, Razvan-Adrian Covache-Busuioc, Petrinel Mugurel Rădoi, Christian-Adelin Covlea, Andrei Adrian Popa, David-Ioan Dumitrascu, Alexandru Vlad Ciurea

**Affiliations:** 1 Department of Neurosurgery, “Carol Davila” University of Medicine and Pharmacy, 020021 Bucharest, Romania; corneliu.toader@umfcd.ro (C.T.); razvan-adrian.covache-busuioc0720@stud.umfcd.ro (R.-A.C.-B.); christian-adelin.covlea0720@stud.umfcd.ro (C.-A.C.); andreiadrianpopa@stud.umfcd.ro (A.A.P.); david-ioan.dumitrascu0720@stud.umfcd.ro (D.-I.D.); prof.avciurea@gmail.com (A.V.C.); 2 Department of Vascular Neurosurgery, National Institute of Neurology and Neurovascular Diseases, 077160 Bucharest, Romania; 3 Neurosurgery Department and Scientific, Sanador Clinical Hospital, 010991 Bucharest, Romania

**Keywords:** cognitive impairment, agraphia, elderly, angular gyrus, Gerstmann syndrome

## Abstract

Gerstmann syndrome, alternatively recognized as angular gyrus syndrome, epitomizes a complex cognitive impairment that has ignited substantial scholarly discourse within the realms of neurology and neuropsychology. The syndrome’s original portrayal was, however, changed. It was demonstrated that the manifestation of its symptomatic tetrad, consisting of four main neurological impairments, is not invariably complete and frequently occurs alongside additional cognitive deficits. Furthermore, the precise cerebral localization of Gerstmann syndrome was traditionally posited to be the left angular gyrus; however, studies mention the possible involvement of other eloquent cerebral areas being pathognomonic. This case report delves into the case of an 86-year-old subject who exhibited the quintessential quartet of symptoms initially delineated by Josef Gerstmann, proposing that elderly patients may manifest a predisposition towards presenting the fully characterized symptomatology initially outlined by Gerstmann.

## 1. Introduction

Gerstmann syndrome (Gs) is identified as a cognitive impairment that leads to a constellation of neurological deficits following damage to the left hemisphere of the brain, specifically targeting the left angular gyrus in its transition to the second occipital convolution [1]. This pathology was first mentioned in 1924 by an Austrian neurologist, Josef Gerstmann, who observed it in a few patients. Gerstmann noted a tetrad of symptoms: finger agnosia, agraphia, acalculia, and right–left disorientation [2,3].

The prevalence of Gerstmann syndrome is challenging to estimate due to a lack of sufficient published cases. Gerstmann himself reported two additional cases in 1927, initially identifying symptoms of finger agnosia and agraphia, and later expanding the syndrome’s definition to include left–right disorientation and acalculia. The term “Gerstmann Syndrome” has been in usage since at least 1934. It is alternatively referred to as “angular gyrus syndrome” or, less frequently, as “Gerstmann Badal syndrome.” In children who appear normal but have brain damage and present with learning disabilities, this condition is referred to as developmental Gerstmann’s syndrome [4].

The cause of Gerstmann syndrome is linked to specific conditions that affect the posterior lobule of the parietal lobe. Various studies have highlighted etiologies such as ischemic stroke [5], brain tumors compressing the angular gyrus [6,7], progressive multifocal leukoencephalopathy [8], and the possibility of acquiring the disease through carbon monoxide poisoning [9]. Furthermore, reports suggest that paroxysmal Gerstmann syndrome might be a manifestation of epilepsy [10,11].

This study aims to explore the presentation of Gerstmann syndrome in an elderly patient, focusing on whether older individuals might be more likely to exhibit the complete tetrad of symptoms originally described by Josef Gerstmann. Additionally, it seeks to investigate the evolving understanding of the syndrome’s cerebral localization, questioning the traditional attribution to the left angular gyrus and considering the possible involvement of other brain regions.

## 2. Case Presentation

An 86-year-old female patient, known with type 2 diabetes mellitus, was admitted to our clinic for hemiparesis on the right side and suggestive signs of Gerstmann syndrome in the last 3 weeks.

Neurological examination presented right-sided hemiparesis MRC 3/5 and cognitive impairments. Specifically, the patient presented finger agnosia, agraphia, acalculia, and right–left disorientation, suggestive manifestations of Gerstmann syndrome.

The intracranial chronic subdural hematoma in this patient is attributed to several factors commonly associated with elderly patients. Age-related cerebral atrophy, which was also observed in the patient’s brain imaging, can lead to an increased vulnerability of the bridging veins, making them more prone to rupture, even with minor trauma. This may explain the development of the chronic subdural hematoma. Although the exact inciting event was not reported by the patient, the gradual onset of symptoms such as hemiparesis and cognitive deficits, including those indicative of Gerstmann syndrome, aligns with the slow progression typical of chronic subdural hematomas. The location of the hematoma, affecting the left hemisphere, including the parietal lobe, further supports its role in the emergence of the cognitive and neurological symptoms observed in this case.

**Figure 1:**
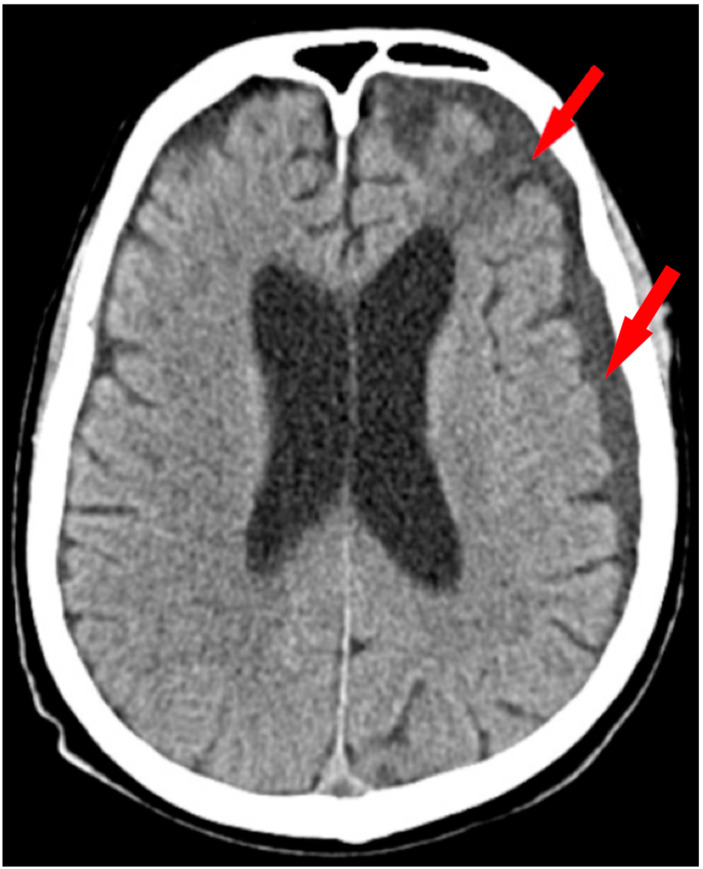
Non-contrast CT scan of the hematoma. Axial section of tissular window CT scan highlights a fronto-parietal chronic subdural hematoma on the left side (red arrow).

A non-contrast brain CT scan revealed a chronic subdural hematoma, Markwalder grade 2, located on the left hemisphere, at the level of frontal and parietal lobes, depicting a hypodense aspect measuring about 1.5 cm, without mass effect or subfalcine herniation. Moderate age-related cerebral abiotrophy was also observed (Figure 1).

**Figure 2:**
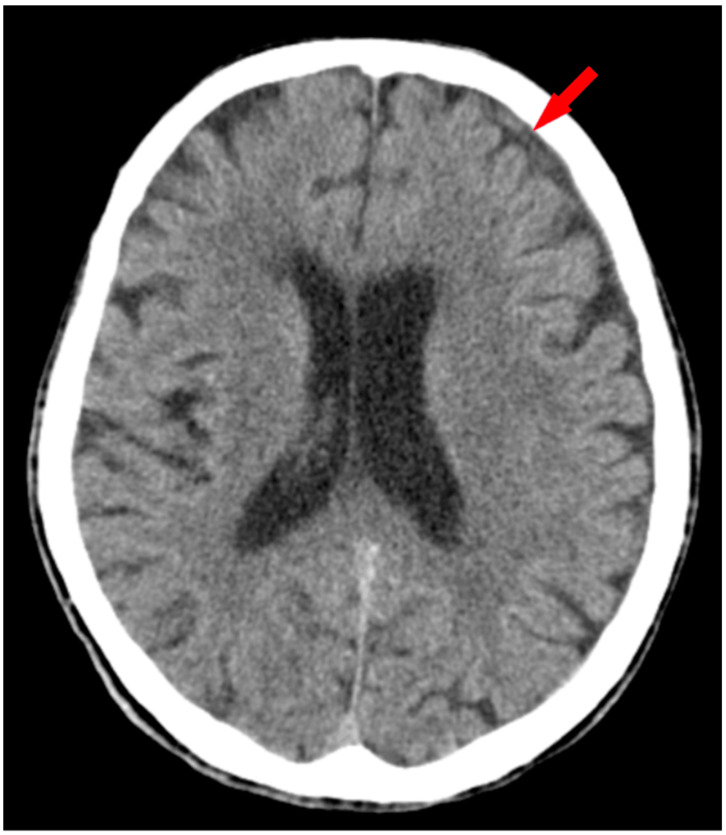
Non-contrast CT scan after hematoma resorption. Axial section of non-enhanced CT examination depicts the subdural hematoma’s significant remission (red arrow).

Computer tomography examination on the next day revealed signs of resorption and a decrease in the hematoma’s thickness (Figure 2).

Considering the patient’s age, the significant reduction of the hematoma, and especially the signs representative of vascular resorption, surgical intervention was not mandatory. At discharge, the patient showed slightly remitted right hemiparesis MRC 4/5; regarding Gerstmann syndrome presentation, no improvements or worsening of the symptoms was observed, and she was prescribed Fludrocortisone for the intracranial hematoma.

## 3. Discussion

Gerstmann syndrome, a subject of debate in neurology and neuropsychology, is associated with lesions in the left angular and supramarginal gyri. This syndrome involves a combination of four neurological deficits, commonly referred to as a tetrad of symptoms: agraphia, acalculia, right–left disorientation, and finger agnosia [12].

Agnosia is understood as an inability to recognize and interpret external information despite normal sensory acuity. Patients with lesions in the left posterior parietal cortex frequently experience finger agnosia, which is the inability to identify their own fingers, yet their sensation and skilled actions are relatively unimpaired [13].

Agraphia, a deficit in writing ability, and acalculia, an impairment in numerical abilities resulting from brain pathology, are also components of Gerstmann syndrome [3]. Lesions in the angular gyrus are primarily associated with agraphia and acalculia [14].

Since its initial characterization in the 1920s, Gerstmann syndrome often presents as an incomplete tetrad of symptoms or is accompanied by cognitive deficits including aphasia, alexia, apraxia, and some perceptual disorders. For example, Uzuner et al. presented a case of a patient with left angular and supramarginal gyrus infarction in the parietal lobe, which manifested symptoms like alexia and anomic aphasia in addition to the symptoms related to Gerstmann syndrome [15].

Additionally, Sakurai et al. reported that alexia occurs as “angular” alexia only when the lesion involves the adjacent lateral occipital gyri. Therefore, only agraphia occurs in angular gyrus lesions, but both agraphia and alexia occur in lesions extending from the angular gyrus to the middle occipital gyrus [16].

In a study by Zukic et al., the frequency and clinical features of Gerstmann syndrome were analyzed among 194 acute stroke patients. While various combinations of agraphia and acalculia were detected, only two patients presented with a complete tetrad of symptoms characteristic of pure Gerstmann syndrome, suggesting its rarity as a clinical entity [17].

Different cognitive models have been studied to understand Gerstmann syndrome. Ardila et al. [18] proposed that the syndrome results from a disturbance in the ability to verbally mediate some spatial knowledge, which impacts both linguistic and numerical operations. This revised conceptualization of Gerstmann syndrome includes acalculia, finger agnosia, right–left disorientation, and semantic aphasia. Agraphia, however, remains unexplained by this unifying mechanism. Nevertheless, agraphia can be observed when the pathology extends toward the superior parietal gyrus [18].

Controversies also exist regarding the exact brain localization of Gerstmann syndrome. While there is a consensus on its association with left hemisphere pathology, the specific brain areas involved remain a subject of debate. Interestingly, there is only one documented case in the available literature describing Gerstmann syndrome associated with a right parietal hemorrhage in an ambidextrous patient [19]. Furthermore, observations indicating that Gerstmann syndrome can manifest during electrical stimulation in the left posterior parietal area further emphasize its angular localization [20].

In the study conducted by João and colleagues, the initial alterations noted in the patient’s neurological assessment were ascribed to disconnection syndrome. This condition was considered a secondary consequence of ischemic damage to the zones of association fibers that facilitate communication between the frontal and parietal lobes. This inference was drawn from the identification of lesions located within the inferior frontal gyrus and insular cortex on the dominant side of the brain. Given the anatomical fact that the middle cerebral artery is responsible for the vascular supply to the insular cortex as well as portions of the frontal and parietal lobes, an alternate explanation proposed for the observed phenomena in this scenario involves the restoration of blood flow to the penumbral region subsequent to clinical intervention [5].

In a separate instance from 2014, a report detailed an elderly patient who developed symptoms of acalculia, agraphia, and confusion between right and left directions—without manifesting finger agnosia—following an ischemic event affecting the left posterior insula and temporal-parietal operculum. Notably, this condition arose in the absence of any damage to the angular and supramarginal gyri. Pathological and functional imaging elucidated connections between the left posterior insular region and the inferior parietal lobe, leading the investigators to theorize that the insular and opercular lesions disrupted essential functional networks, resulting in symptoms mimicking those of parietal lobe syndrome [21].

In 2016, research by Eun-Ju Lee et al. [22] on two patients presenting with clinical manifestations aligning with the Gerstmann syndrome tetrad revealed ischemic lesions in the left medial frontal lobe, conspicuously sparing the angular and supramarginal gyri along with adjacent structures. The authors posited that the observed symptoms could be attributed to a disconnection among the association fibers. Additionally, they emphasized the crucial role of both cortical and subcortical regions in the left frontal lobe in the pathogenesis of Gerstmann syndrome. This significance is underpinned by the robust connections these areas share with the parietal lobe [22]. Recent investigations have highlighted the utility of diverse diagnostic approaches in delineating Gerstmann syndrome. Research by Vaddiparti and colleagues involved the application of Electrical Cortical Stimulation (ECS) mapping on a subject suffering from non-lesional medically intractable epilepsy, aimed at delineating the functional anatomy pertinent to Gerstmann syndrome. This technique pinpointed precise cortical regions linked to each symptom of the syndrome; acalculia was induced by stimulation of the superior parietal lobule and the upper parts of the supramarginal and angular gyri; agraphia was triggered by activation of the superior parietal lobule; left–right confusion was elicited through stimulation of the superior angular gyrus; and finger agnosia, which was only identifiable with the patient’s eyes closed, resulted from stimulating the angular gyrus and the lower segment of the superior parietal lobule. The convergence of all four symptoms was observed near the lower section of the superior parietal lobule, revealing a nuanced interconnection among the various components of Gerstmann syndrome within the dominant parietal cortex, characterized by limited cortical overlap [20].

Additionally, the clinical observations and neuroimaging results from Yoon’s investigation propose that Gerstmann syndrome may manifest as a disconnection syndrome, originating from damage to the white matter pathways that link the inferior parietal cortex to other cerebral regions. The refinement of diffusion tensor imaging (DTI) methodologies has contributed to a growing body of evidence that supports the conceptualization of Gerstmann syndrome as a disconnection syndrome. This is attributed to the interruption of subcortical white matter tracts rather than to lesions situated in cortical areas [23].

The literature contains few cases of Gerstmann syndrome in elderly patients, making it challenging to determine age-related differences in individuals with this condition. The scarcity of such cases emphasizes the need for further investigation into specific features of the disease observed in older demographics (Table 1).

Table 1 indicates that Gerstmann syndrome in elderly patients typically presents as a complete tetrad of symptoms, manifesting irrespective of the individual’s gender. This observation suggests that advanced age could be a significant factor in the full expression of the four distinct symptoms of Gs. Consequently, it might be feasible to categorize Gs based on age-related demographic factors.

Unconventional manifestations of chronic subdural hematomas encompass extrapyramidal symptoms and other infrequent neurological conditions, such as Gerstmann syndrome [29]. The inaugural documentation of chronic subdural hematoma associated with Gerstmann syndrome was provided by Maeshima et al. in 1998 [30]. This case involved a 71-year-old right-handed female who exhibited symptoms of right arm and leg weakness over a two-week duration. Diagnostic imaging, specifically computerized tomography (CT) of the head, disclosed a significant left fronto-parietal, extra-axial hypodense fluid collection interspersed with scattered hyperdense areas. The therapeutic intervention consisted of a left parietal trepanation and subsequent hematoma evacuation. Remarkable clinical improvement was observed three days post-operatively, evidenced by the patient’s ability to write with minimal errors and the resolution of her hemiparesis. Neuropsychological assessments conducted seven days after the surgery also demonstrated significant enhancements [30]. The underlying mechanism remains a subject of considerable debate; the hypotheses of direct compression or the establishment of a hemispheric pressure differential by the chronic subdural hematoma have been considered. However, these explanations are thought to be overly generalized [31].

Currently, the treatment approach for Gs is mainly symptomatic and supportive, focusing on the management of the underlying neurological condition. In cases involving brain injuries or tumors, surgical interventions might be employed to mitigate the symptoms. Nonetheless, ongoing research holds the potential for developing more targeted treatments, which could lead to improved outcomes for patients with Gerstmann syndrome.

While this case report provides valuable insights into the presentation of Gerstmann syndrome in elderly patients, its findings are based on a single case. Future studies should aim to explore the condition in larger cohorts to confirm the trends observed here. Additionally, incorporating advanced imaging techniques, such as functional MRI, could help further clarify the cerebral areas involved and the underlying pathophysiology. These efforts will contribute to a more comprehensive understanding of Gerstmann syndrome, particularly in elderly populations.

## 4. Conclusions

To conclude, Gerstmann syndrome continues to be a subject of considerable debate within the scientific literature. The manifestation of this syndrome has varied over the years, diverging from the initial symptoms reported. Currently, the advent of advanced diagnostic methodologies has yielded auspicious outcomes, heralding new avenues for exploration and research in this domain.

Moreover, the knowledge presents a discernible lacuna regarding the epidemiological and demographic profiling of Gerstmann syndrome. Therefore, additional case studies have to be conducted, especially for an in depth understanding of the possibility of a propensity among older individuals to manifest the comprehensive symptomatology originally identified. This observation is enforced by our case study of an 86-year-old patient, who exhibited all the hallmark symptoms of Gerstmann syndrome, and additionally, an atypical presentation of Gerstmann syndrome in the context of a chronic subdural hematoma, thereby enriching the corpus of knowledge and contributing significantly to the nuanced understanding of this neurological condition.

## Figures and Tables

**Table 1:** Various studies of Gerstmann syndrome (GS) in elderly patients.

First Author & Year	N	Age	Gender	Etiology for GS	Neurological Signs
Turconi et al 2002 [24]	1	73	M	Left posterior parietal cerebrovascular stroke	Agraphia, acalculia, right-left disorientation, finger agnosia
Kim et al 2003 [25]	1	77	F	Intracerebral lobar hemorrhage at the left occipito-parietal area	Agraphia, acalculia, right-left disorientation, finger agnosia
Chen et al 2013 [26]	1	72	M	Acute cerebral infarction involving the parietal lobe	Agraphia, acalculia, right-left disorientation, finger agnosia
Barbosa et al 2017 [27]	1	75	F	Diffuse atheromatosis in the left middle cerebral artery	Agraphia, acalculia, right-left disorientation, finger agnosia
Aguirre et al 2022 [28]	1	79	F	Breast cancer in remission—one tumor lesion in the angular gyrus of the parietal cortex	Agraphia, acalculia, right-left disorientation, finger agnosia

N = number of patients; M = male; F = female; GS = Gerstmann syndrome

## Data Availability

The data presented in this study are available on request from the corresponding author.

## References

[1] Basagni B., Luzzatti C., De Tanti A., Bozzetti F., Crisi G., Pinardi C., Errante A., Fogassi L. (2021). Some evidence on Gerstmann’s syndrome: A case study on a variant of the clinical disorder. Brain Cogn..

[2] Cubelli R., Rusconi E. (2022). The making of a syndrome: Gerstmann’s patients before Gerstmann syndrome. Cortex.

[3] Ardila A. (2020). Gerstmann Syndrome. Curr. Neurol. Neurosci. Rep..

[4] Orlova N., Shkliar M., Tarasov V. (2021). Gerstmann Syndrome in Adult Ukrainian Woman. Psychosom. Med. Gen. Pract..

[5] João R.B., Filgueiras R.M., Mussi M.L., de Barros J.E.F. (2017). Transient Gerstmann syndrome as manifestation of stroke: Case report and brief literature review. Dement. Neuropsychol..

[6] Kleinschmidt A., Rusconi E. (2011). Gerstmann meets Geschwind: A crossing (or kissing) variant of a subcortical disconnection syndrome?. Neuroscientist.

[7] Feller C., Kelly N. (2021). A-62 Case of Gerstmann’s Syndrome Due to Brain Tumor of Unknown Etiology. Arch. Clin. Neuropsychol..

[8] Sanjo N., Kina S., Shishido-Hara Y., Nose Y., Ishibashi S., Fukuda T., Maehara T., Eishi Y., Mizusawa H., Yokota T. (2016). Progressive Multifocal Leukoencephalopathy with Balanced CD4/CD8 T-Cell Infiltration and Good Response to Mefloquine Treatment. Intern. Med..

[9] Kato M. (2015). Carbon monoxide poisoning: Clinical features of the victims of the explosion accident of Mitsui- Miike Mikawa coal mine 50 years ago. Brain Nerve.

[10] Khoo H.M., Fujita Y., Tani N., Oshino S., Kagitani-Shimono K., Kishima H. (2020). Mystery Case: Parietal lobe epilepsy with ictal manifestation of Gerstmann syndrome. Neurology.

[11] Zöllner J.P., Haag A., Hermsen A., Bauer S., Stahl F., Wulf K., Menzler K., Reif P.S., Wagner M., Pagenstecher A. (2017). Ictal conduction aphasia and ictal angular gyrus syndrome as rare manifestations of epilepsy: The importance of ictal testing during video-EEG monitoring. Epilepsy Behav. Case Rep..

[12] Rusconi E. (2018). Gerstmann syndrome: Historic and current perspectives. Handb. Clin. Neurol..

[13] Tamè L., Dransfield E., Quettier T., Longo M.R. (2017). Finger posture modulates structural body representations. Sci. Rep..

[14] Al-Samaraie A.A.S. (2023). Gerstmann Syndrome Case-Control Study Correlation between Brain Lesions & Functional Disability. Int. Tinnitus J..

[15] Uzuner G.T., Ubur A., Erten M., Uzuner N. (2020). A Rare Clinical Antity; Pure Gerstmann Syndrome. J. Stroke Cerebrovasc. Dis..

[16] Sakurai Y., Asami M., Mannen T. (2010). Alexia and agraphia with lesions of the angular and supramarginal gyri: Evidence for the disruption of sequential processing. J. Neurol. Sci..

[17] Zukic S., Mrkonjic Z., Sinanovic O., Vidovic M., Kojic B. (2012). Gerstmann’s Syndrome in Acute Stroke Patients. Acta Inform. Med..

[18] Ardila A. (2014). A proposed reinterpretation of Gerstmann’s syndrome. Arch. Clin. Neuropsychol..

[19] Nicastro N., Tafer N., Schnider A., Di Pietro M. (2017). Gerstmann’s Syndrome Associated with Right Parietal Hemorrhage and Arteriovenous Malformation. J. Clin. Neurol..

[20] Vaddiparti A., McGrath H., Benjamin C.A.F., Sivaraju A., Spencer D.D., Hirsch L.J., Damisah E., Quraishi I.H. (2021). Gerstmann Syndrome Deconstructed by Cortical Stimulation. Neurology.

[21] Bhattacharyya S., Cai X., Klein J.P. (2014). Dyscalculia, Dysgraphia, and Left-Right Confusion from a Left Posterior Peri-Insular Infarct. Behav. Neurol..

[22] Lee E.J., Shin H.Y., Noh Y., Park K.H., Park H.M. (2016). Two Cases with Cerebral Infarction in the Left Middle Frontal Lobe Presented as Gerstmann’s Syndrome. Neurol. Disord..

[23] Yoon S.H., Lee J.I., Kang M.J., Lee H.I., Pyun S.-B. (2023). Gerstmann Syndrome as a Disconnection Syndrome: A Single Case Diffusion Tensor Imaging Study. Brain Neurorehabil..

[24] Turconi E., Seron X. (2002). Dissociation Between Order and Quantity Meanings in a Patient with Gerstmann Syndrome. Cortex.

[25] Kim N.R., Chung J.G., Lee S.K. (2003). Cerebral Amyloid Angiopathy: A Case Report. J. Pathol. Transl. Med..

[26] Chen T.Y., Chen C.Y., Yen C.H., Kuo S.C., Yeh Y.W., Chang S., Huang S.Y. (2013). Acute parietal lobe infarction presenting as Gerstmann’s syndrome and cognitive decline mimicking senile dementia. Neuropsychiatr. Dis. Treat..

[27] Barbosa B.J.A.P., de Brito M., Rodrigues J.C., Kubota G.T., Parmera J.B. (2017). Gerstmann’s syndrome and unilateral optic ataxia in the emergency department. Dement. Neuropsychol..

[28] Orozco Aguirre A.L., Valdovino M.M., Santos M.A., Meléndez A.M. (2022). Secondary Gerstmann syndrome, a case report. Eur. Psychiatry.

[29] Bhatt P.M. (2023). Chronic Subdural Hematoma: Past, Present, and Future. Indian J. Neurotrauma.

[30] Maeshima S., Okumura Y., Nakai K., Itakura T., Komai N. (1998). Gerstmann’s syndrome associated with chronic subdural haematoma: A case report. Brain Inj..

[31] Symon L., Pasztor E., Dorsch N.W.C., Branston N.M. (1973). Physiological Responses of Local Areas of the Cerebral Circulation in Experimental Primates Determined by the Method of Hydrogen Clearance. Stroke.

